# Clinical Audiological Features of Otosclerosis—Preoperative Hearing Analysis in 80 Ears

**DOI:** 10.1007/s12070-025-05586-2

**Published:** 2025-06-06

**Authors:** Junfang Xue, Jianjun Sun

**Affiliations:** Department of Otolaryngology-Head and Neck Surgery, Peking University International Hospital, No.1 Life Park Road, Zhongguancun Life Science Park, Changping District, Beijing, 100026 China

**Keywords:** Otosclerosis, Willis paracusis, Schwarz sign, Gellé test, Carhart notch

## Abstract

The incidence of otosclerosis is lower in yellow and black populations compared to white populations. Preoperative diagnosis and surgical indications are based on clinical features and audiological evaluations. To summarize the clinical features and analyze audiological characteristics of otosclerosis in China, aiming to improve the diagnosis and determination of surgery given its low incidence in the country. A retrospective analysis was conducted involving 80 ears (48 patients) who underwent surgery for otosclerosis from 2003 to 2023. Among the patients, 88.8% experienced tinnitus, only 2.5% had Willis paracusis, Schwarz sign was all absent, and Gellé test was negative in 98.7%. Furthermore, 31.2% of the patients did not exhibit a Carhart notch, while 63.6% had a Carhart notch at 2 kHz, and 5.2% at 1 kHz. Tinnitus is the main accompanying symptom of otosclerosis. Willis Paracusis and Schwarz sign are relatively rare in otosclerosis. The Gellé test provides reliable diagnostic criteria for otosclerosis. The presence of a Carhart notch is not definitive for diagnosing otosclerosis and does not appear exclusively at 2 kHz. Significance: P < 0.05.

## Introduction

Otosclerosis is a primary disease characterized by impaired metabolism within the bony labyrinth. It begins with softening of osseous tissue, followed by secondary pathological sclerosis. The disease usually involves the anterior niche of the oval window, which induces the stapes to be fixed. Otosclerosis is a common cause of conductive hearing loss with an intact tympanic membrane. Sensorineural hearing loss may also occur if otosclerotic foci develop within the cochlea.

The diagnosis of otosclerosis involves comprehensive assessment, including medical history, hearing tests, and imaging. Distinguishing otosclerosis from other conditions remain a diagnostic challenge. Surgery can ultimately confirm the diagnosis of otosclerosis and is an effective treatment method for improving hearing. Nevertheless, preoperative diagnosis and determination of surgical indications are particularly important.

Otosclerosis is common in Europe, the Middle East, North America, South America and India. However, its prevalence in China is much lower. In this study, we reviewed the literature and summarized the incidence of otosclerosis worldwide. We retrospectively investigated the clinical and audiological characteristics of 80 ears with otosclerosis in China.

## Materials and Methods

The approval of the bioethics committee for the study was obtained. A retrospective analysis was conducted on 80 ears (48 patients) that underwent surgery in Peking University International Hospital between 2003 and 2023. The mean age was 38.41 ± 12.34 years, ranging from 11 to 58 years. Among the patients, there were 36 women and 12 men.

All cases were confirmed through surgery and underwent laser-assisted stapedotomy. A detailed clinical history, physical examination, imaging studies, and audiometric testing were conducted for all patients. Preoperative audiograms were obtained one week before surgery. All audiometric tests were performed by certified clinical audiologists using an AD229b audiometer (Interacoustics, Denmark).

The distribution of Carhart notches at different frequencies was analyzed. Carhart notch was defined as an impairment in the BC of ≥ 7.5 dB above the higher and lower adjacent frequencies.

Statistical analyses were performed using SPSS 20.0. The chi-square test was used to assess statistically significant differences among the Carhart notch groups. Statistical significance was set at *P* < 0.05.

## Results

Table 1 presents the clinical manifestations of otosclerosis in the 80 ears. The male-to-female ratio was 1:3, with a predominance of middle-aged patients (mean age, 38years). Among the 48 patients, 32 had bilateral onset while 16 had unilateral onset. Bilateral involvement was noted in 66.7% of the cases. Tinnitus was present in 88.8% of patients, while only 10% experienced ear fullness. Willis paracusis was observed in 2.5% of patients, Schwarz sign was absent, and the Gellé test was negative in 98.7% of cases. Increased stapes density and decreased bone density in the vestibular window area were noted in 19.7% of patients. Additionally, 19.7% of the patients exhibited a type As tympanogram.

**Table 1:** Clinical manifestations of otosclerosis in 80 ears

Clinical manifestations	positive rate(%)	negative rate(%)
Gender	75%(36/48) female	25%(12/48)male
Age	38.41 ± 12.34y	
Side	66.7%(32/48) bilateral	33.3%(16/48) lateral
Tinnitus	88.8% (51/80)tinnitus in different degrees	11.2%(29/80)no tinnitus
Ear stuffy	10%(8/80) ear stuffy	90% (72/80)no stuffy
Willis paracusis	2.5%(2/80) paracusis	97.5% (78/80)no paracusis
Schwarz Sign	None	
Gellé test	1.3%(1/80)+	98.7% (79/80)-
Tympanogram	19.7%(15/76) type As	80.2%(61/76) type A
Carhart Notch	5.2%(4/77) in 1 kHz 63.6%(49/77) in 2khz 31.2%(24/77) no notch	
Image	19.7%(13/66) positive	80.3%(53/66) negative

**Fig. 1:**
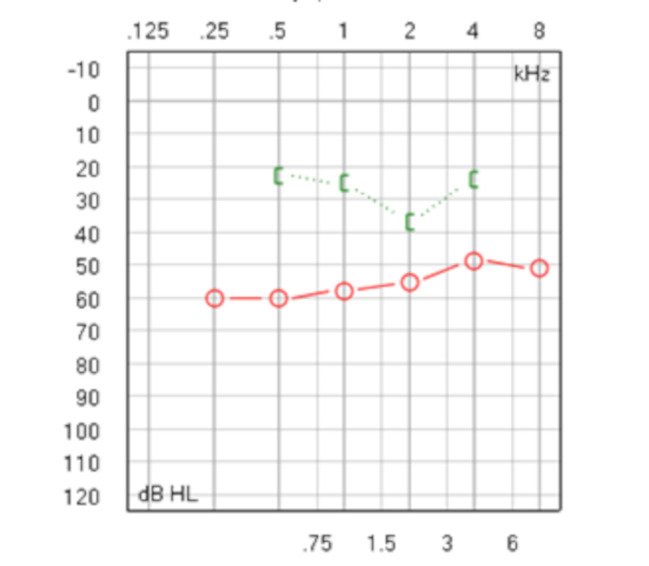
The average pure-tone audiometry of 80 ears with otosclerosis

Table 2 and Fig. 1 display the pure-tone audiometry results for 80 ears. The average air conduction threshold (AC) ranged from 49 to 60 dB HL, whereas the bone conduction threshold (BC) averaged between 22- and 36 dB HL. As the test frequency increased, the AC decreased gradually and increased slightly at 8 kHz. In contrast, BC gradually increased with increasing test frequency and decreased at 4 kHz.

**Table 2:** PTA of 80 ears with otosclerosis

Frequency(Hz)	AC(dB HL)	BC(dB HL)	ABG(dB HL)
250	60.19 ± 10.74	-	-
500	60.19 ± 10.02	22.56 ± 8.78	37.73 ± 8.98
1k	58.21 ± 11.65	25.39 ± 10.38	32.86 ± 10.46
2k	54.49 ± 13.33	36.56 ± 11.19	17.86 ± 8.25
4k	49.04 ± 18.38	24.35 ± 15.65	24.61 ± 12.08
8k	50.87 ± 19.61	-	-

AC: air conduction; BC: bone conduction; ABG: air–bone gap

**Fig. 2:**
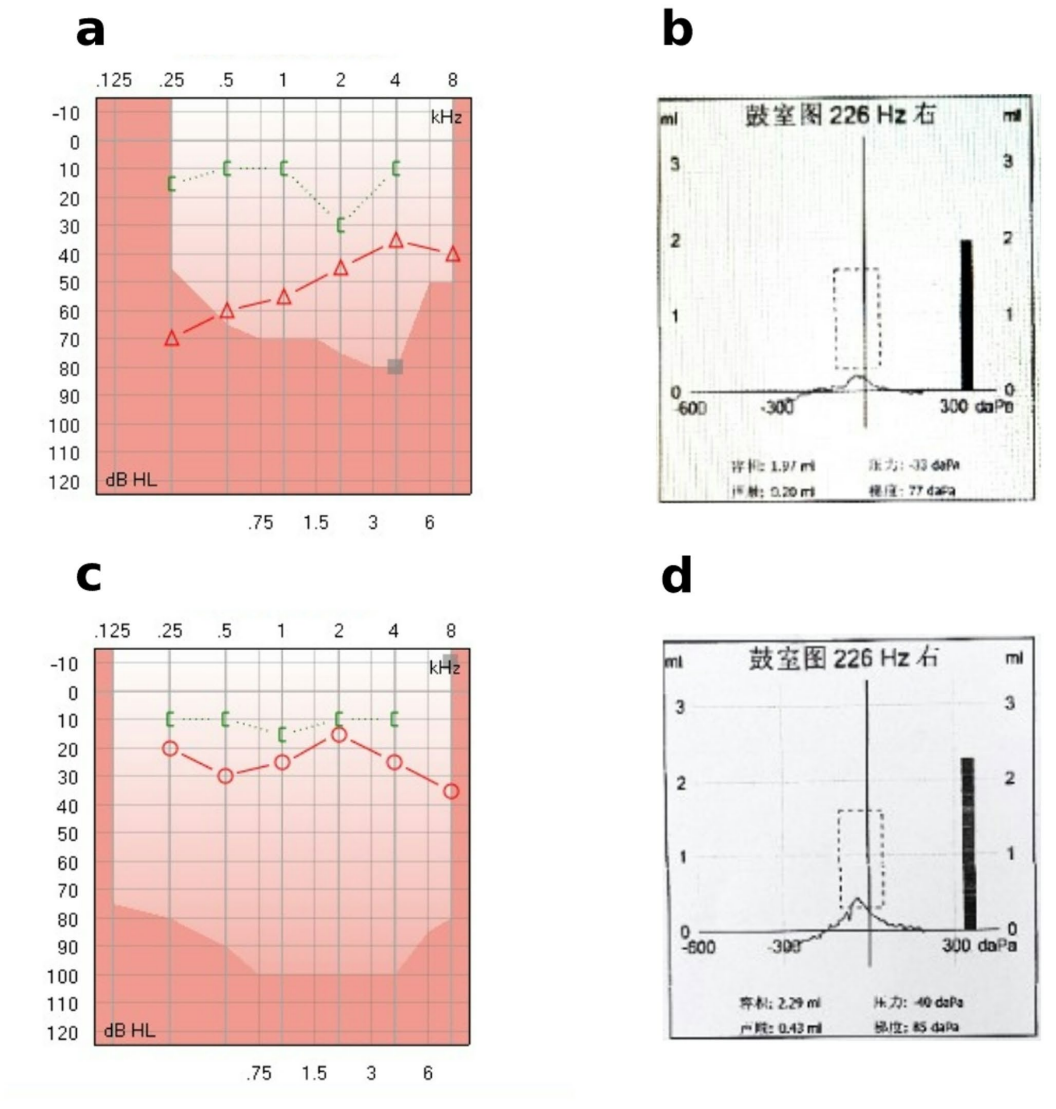
Preoperative and postoperative hearing performance of classic case (**a**. preoperative audiogram showed conductive deafness of low-tone more severe, and Carhart Notch was presented at 2 kHz; **b**. preoperative tympanogram showed type As; **c**. closing the ABG after surgery; **d**. tympanogram became type A after surgery)

In this study, 31.2% of the patients had no Carhart notch, 63.6% presented Carhart notch at 2 kHz, and 5.2% at 1 kHz. The presence of the notch did not correlate with the hearing threshold (Table 3; Fig. 2).

**Table 3:** The distribution of the Carhart notch

Carhart	*n*	AC(dB HL)	BC(dB HL)	ABG(dB HL)
No Carhart	24	55.26 ± 12.60	28.23 ± 10.96	27.03 ± 7.13
1 kHz Carhart	4	55.31 ± 13.75	34.06 ± 5.04	21.25 ± 8.72
2 kHz Carhart	49	55.40 ± 11.33	26.12 ± 8.75	29.22 ± 7.00
P		0.878	0.613	0.643

## Discussion

The prevalence of otosclerosis is influenced by race, age, and gender. In particular, otosclerosis is more common among white individuals, whereas its occurrence is lower in yellow and black populations. Table 4 shows the prevalence of otosclerosis among the different populations over the past 40 years. According to a comprehensive report from 2011, the overall prevalence of otosclerosis in the United States is 20 cases per 100,000 individuals. Among Hispanics, Whites, and African Americans, the rates were 43, 12.6, and 3 per 100,000, respectively [1]. Another long-term epidemiological study conducted in the United States reported changes in otosclerosis incidence between 1950 and 2017. It showed that the incidence of otosclerosis peaked in the early 1970s at 18.5 per 100,000 people, and then declined significantly, dropping to 3.4 per 100,000 people from 2015 to 2017 [2]. Between 2011 and 2017, a study in the UK involving 657 otosclerosis patients found that 85% were White/White British, 7% were Asian/Asian British, and 3% were Black/Black British [3]. However, a study in Japan reported a significantly lower prevalence of otosclerosis among the population, at 1.48% for histological otosclerosis [4]. Notably, there have been no reports on the epidemiology of otosclerosis in China.

**Table 4:** Prevalence of otosclerosis in the different population

Race	Country	time	Prevalence	Sample size	Diagnostic bases	Author
White	Sweden	1981	6.1/1,000,000	—	clinical	Levin [28]
White	South Australia	1984	Males 0.5%, Females 1%	4019	clinical	Gristwood [29]
White	AmericanMinnesota	1991	12.75%	1452	histologic	Hueb [30]
White	Danmark	1999	0.13–0.15%	27,692	clinical	Parving[31]
White	Tunisia	2001	0.4–0.8%	—	clinical	Ben [32]
White	UK	2001	2.5% 0.3-0.38%	236	histologic clinical	Declau[33] extrapolated
White	American	2011	20/100,000	672,839	clinical	Choi [1].
Yellow	Japanese	2003	1.48%	1011	histologic	Ohtani [4]
Black	South African	2005	15/156,000	156,000	clinical	Tshifularo[34]

Due to the different prevalence of otosclerosis worldwide, otosclerosis should be considered a priority diagnosis for conductive hearing loss patients with normal intact tympanic membranes in high-incidence regions. Corresponding healthcare institutions may increase screening for earlier detection; for example, in Europe and America, regular hearing tests may focus on individuals with a family history or at high risk [3]. Conversely, in East Asia or Africa, additional diagnostic evidence may need to be gathered before confirming the diagnosis, and screening may rely more on patient-initiated visits.

Otosclerosis predominantly affects women, and this correlation is often attributed to estrogen levels. It typically manifests bilaterally and involves late-onset, progressive hearing loss. Clinically, the male-to-female ratio ranges from 1:2 to 1:1.5. The male-to-female ratio in this study was 1:2.8, and the age of onset was 38 + years, which is consistent with previous findings.

In this study, 88.8% of patients complained of tinnitus. Gristwood and Venables [5] reported that 65% of participants were diagnosed with chronic tinnitus before surgery among 1,014 patients with otosclerosis. In a study of 157 otosclerosis patients reported by Skarzynski [6], 68.2% accompanied by tinnitus, which was not correlated with the degree of hearing loss. Tinnitus was the main accompanying symptom of otosclerosis.

In 1672, Dr. Thomas Willis described a peculiar phenomenon in two people with hearing loss who appreciated some recovery of hearing in the presence of increased background noise. Over time, this clinical entity came to bear its name, Willis Paracusis. This phenomenon has been listed as a diagnostic criterion for otosclerosis [7]. However, some are plausible by today’s understanding, while many do not. In this study, only 2 individuals reported experiencing Willis Paracusis. Therefore, Willis Paracusis cannot be used as a diagnostic criterion for otosclerosis.

Schwarz sign refers to mucosal congestion of the promontorium tympani observed through the tympanic membrane. None of the 80 cases in this study exhibited Schwarz sign. Hao XP [8] reported 200 patients with otosclerosis, none of whom exhibited Schwarz sign. So far, there has been only case report among patients with otosclerosis [9, 10]. Scholars believe that Schwarz is rare and cannot be used as a diagnostic criterion for otosclerosis [11].

In 1885, Gellé included a negative Gellé test as a diagnostic criterion for otosclerosis [12]. The negative rate of Gellé reported by Hao XP [8] was 92.09%, and in this study the negative rate of Gellé reached 98.7%. These findings highlight the Gellé test can be a reliable diagnostic standard for otosclerosis.

In this study, the tympanogram was type A in 80.2% of the patients, and type As in 19.7%. Among the 107 ears of otosclerosis reported by Kan [13], only one ear showed a type B tympanogram, the other ear showed type C, and the remaining ears showed type A. These findings indicate that A tympanograms predominate in patients with otosclerosis, with type As representing a minority. It is speculated that tympanometry may reflect more middle ear lesions, but is not sensitive to ossicular chain activity.

According to Table 2 and Fig. 1, the air conduction is more severe at low frequencies, and the air-bone gap is up to 40 dB. However, the decrease in bone conduction was not pronounced, and the most significant decrease was observed at 2 kHz, indicative of the Carhart notch. Hearing loss in patients with otosclerosis reflects the disease progression. Genc [14] suggested that when the preoperative air-bone gap exceeds 40 dB, diffuse otosclerosis should be considered, as the condition may involve more than just the stapes ring ligament, potentially affecting the postoperative hearing outcomes.

In this study, the incidence of Carhart notch in otosclerosis was 68.8%, consistent with the 31-80% reported in the literature. The Carhart notch was originally described by Carhart in 1950, emphasizing its prominence at 2 kHz. In 1978, Ginsberg [15] analyzed the hearing of 2, 405 stapedectomies in detail and found that bone conduction improvements were roughly equal between 0.5 and 2 kHz. Gatehous [16] believed that the increase in bone conduction occurred in the frequency range of 500-2,000 Hz and renamed it the “Carhart effect”. Lamblin [17] reviewed the hearings of 1029 otosclerosis surgery patients and noted that Carhart exhibited a wider range of frequencies, including 29.2% at 2 kHz, 4.7% at 1 kHz, and 19.1% at 0.5 kHz. Similar to our study, the Carhart notch manifests across a spectrum of frequencies, and not exclusively at 2 kHz.

Carhart notch is observed not only in otosclerosis but also in other middle ear diseases that affect ossicular chain movement. Kashio [18] reported no statistically significant difference in the incidence and depth of the Carhart notch between stapes fixation in otosclerosis and ossicular chain malformation. Yasan [19] reviewed 315 patients undergoing surgery for middle ear lesions and found that Carhart notch was present in tympanosclerosis (19.6%), chronic otitis media (12.4%), secretory otitis media (7.1%), ossicular anomalies (50%), and other diseases. Therefore, the Carhart notch serves as a valuable indicator in the diagnosis of otosclerosis; however, it should not be employed as a definitive diagnostic criterion.

The Carhart notch represents the progression of otosclerosis to some extent. Job [20] compared the preoperative and postoperative hearing of 116 patients with otosclerosis and found that the Carhart notch was an adverse factor affecting postoperative hearing improvement. Wiatr [21] retrospectively analyzed 140 patients who underwent initial surgery for otosclerosis between 2010 and 2016 and found that the presence of the Carhart notch was an adverse prognostic factor for the improvement of bone conduction and ABG. Lamblin [17] retrospectively compared the hearing of 931 patients undergoing otosclerosis surgery, and also believed that Carhart notch was a negative factor for postoperative bone conduction hearing improvement. According to a previous study, the Carhart effect is due to the lack of excitation mainly from the inertial component of the ossicles and to a lesser extent from the inertia of the cochlear fluid [22].

The management of otosclerosis includes sodium fluoride therapy, surgical intervention, and hearing aids [23]. Vincent [24] reviewed 3,050 cases of stapedotomy and found that surgery was safe and successfully treated 94.2% of patients with conductive hearing loss. However, because of the progressive characteristics of this disease, 10–20% of patients are likely to need revision surgery [25]. A study in France reported that treating otosclerosis surgically was slightly less expensive than hearing aids for over 10 years [26]. The clinical therapy features also vary regionally. For example, in some Caucasus countries, patients may present with more severe symptoms at a younger age, prompting earlier and more aggressive surgical approaches [27]. In contrast, in Asia, symptoms may be milder and progress more slowly, allowing for more conservative management strategies.

## Conclusion

The incidence of otosclerosis is lower in yellow and black populations than in white populations. Tinnitus is the main accompanying symptom of otosclerosis. Willis Paracusis and Schwarz sign are are relatively rare in otosclerosis. The Gellé test provides reliable diagnostic criteria for otosclerosis. The presence of a Carhart notch is not definitive for diagnosing otosclerosis and does not appear exclusively at 2 kHz.

These findings provide valuable insights into the clinical characteristics and audiological features of otosclerosis in a Chinese population. Further research is required to validate and expand upon these results. Improved diagnostic methods can contribute to earlier and more accurate diagnoses of otosclerosis, ultimately leading to improved treatment outcomes.
